# PANDEMIC-SPECIFIC SUPPORTS AND CHALLENGES: ASSOCIATIONS WITH DEMENTIA CAREGIVER WELL-BEING

**DOI:** 10.1093/geroni/igac059.473

**Published:** 2022-12-20

**Authors:** Amanda Leggett, Hyun Jung Koo, Cathleen Connell, Laura Gitlin, Helen Kales

**Affiliations:** Wayne State University, Ypsilanti, Michigan, United States; University of Michigan, Ann Arbor, Michigan, United States; University of Michigan, Ann Arbor, Michigan, United States; Drexel University, Philadelphia, Pennsylvania, United States; University of California, Davis, Davis, California, United States

## Abstract

This study examines the prevalence of pandemic-specific care supports and challenges (e.g., increased support from family and friends, difficulty accessing respite care, confusion on public health guidelines) and associations with stress and well-being among 100 family caregivers for persons living with dementia interviewed in 2021. Pandemic care challenges were common- 52% reported a decrease in support from family and friends, 43% had difficulty accessing medical care, and 31% had difficulty getting needed in-home and out-of-home services. Accounting for demographics and the care context, difficulties accessing various types of respite care (e.g., paid respite care, respite from family/friends) were associated with caregiver stress, burden, and less positive affect. Pandemic supports, including increased support from family and friends and receiving information on COVID-care were associated with greater positive affect, but not caregiver stress. While care-supports enhanced well-being, efforts to help caregivers take breaks from caregiving may have significant impact on reducing stress.

